# Supplementary material for: Musculoskeletal surgeons have implicit bias towards the biomedical paradigm of human illness
Source: PLoS One. 2024 Oct 24;19(10):e0310119. doi: 10.1371/journal.pone.0310119 (PMC11500957; doi:10.1371/journal.pone.0310119)
Supplement: Attachment — Submitted filename: PONE-D-24-08426.pdf [file pone.0310119.s002.pdf]

PLOS ONE

Musculoskeletal Surgeons Have Implicit Bias Towards the Biomedical Paradigm of Human Illness

--Manuscript Draft--

**Manuscript Number:**	PONE-D-24-08426
**Article Type:**	Research Article
**Full Title:**	Musculoskeletal Surgeons Have Implicit Bias Towards the Biomedical Paradigm of Human Illness
**Short Title:**	Musculoskeletal Surgeons Have Implicit Bias Towards the Biomedical Paradigm of Human Illness
**Corresponding Author:**	David Ring, MD PhD<br>The University of Texas at Austin Dell Medical School<br>Austin, UNITED STATES
**Keywords:**	implicit bias, surgeon bias, Implicit attitude test, biomedical, biopsychosocial
**Abstract:**	Background<br><br>Evidence is mounting that the biopsychosocial paradigm is more accurate and useful than the biomedical paradigm of care. Habits of thought can hinder the implementation of this knowledge into daily care strategies. To understand and lessen these potential barriers, we asked: 1) What is the relative implicit and explicit attitudes of musculoskeletal surgeons towards the biomedical or biopsychosocial paradigms of medicine? 2) What surgeon factors are associated with these attitudes?<br><br>Methods<br><br>An online survey-based experiment was distributed to members of the Science of Variation Group (SOVG) with a total of 163 respondents. Implicit bias towards the biomedical or biopsychosocial paradigms was measured using an Implicit Association Test (IAT) developed by our team using open-source software; explicit preferences were measured using ordinal scales.<br><br>Results<br><br>On average, surgeons demonstrated a moderate implicit bias towards the biomedical paradigm (d-score: -0.21; Interquartile range [IQR]: -0.56 to 0.19) and a moderate explicit preference towards the biopsychosocial paradigm (mean: 14; standard deviation: 14). A greater implicit bias towards the biomedical paradigm was associated with male surgeons (d-score: -0.30; IQR: -0.57 to 0.14; P = 0.005). A greater explicit preference towards the biomedical paradigm was independently associated with a European practice location (Regression coefficient: -9.1; 95% CI: -14 to -4.4; P <0.001) and trauma subspecialty (RC: -6.2; 95% CI: -11 to -1.0; P <0.001).<br><br>Conclusions<br><br>The observation that surgeons have an implicit bias favoring the biomedical paradigm might inform strategies for implementation of care strategies based on evidence favoring the biopsychosocial paradigm.
**Order of Authors:**	Sina Ramtin
	Dayal Rajagopalan
	David Ring, MD PhD
	Tom Crijns
	Prakash Jayakumar
**Additional Information:**	

Powered by Editorial Manager® and ProduXion Manager® from Aries Systems Corporation

Question	Response
**Financial Disclosure**<br><br>Enter a financial disclosure statement that describes the sources of funding for the work included in this submission. Review the submission guidelines for detailed requirements. View published research articles from *PLOS ONE* for specific examples.<br><br>This statement is required for submission and **will appear in the published article** if the submission is accepted. Please make sure it is accurate.<br><br>**Funded studies**<br>Enter a statement with the following details:<br>• Initials of the authors who received each award<br>• Grant numbers awarded to each author<br>• The full name of each funder<br>• URL of each funder website<br>• Did the sponsors or funders play any role in the study design, data collection and analysis, decision to publish, or preparation of the manuscript?<br><br>Did you receive funding for this work?	The author(s) received no specific funding for this work.
**Competing Interests**<br><br>Use the instructions below to enter a competing interest statement for this submission. On behalf of all authors, disclose any competing interests that could be perceived to bias this work—acknowledging all financial support and any other relevant financial or non-financial competing interests.<br><br>This statement is required for submission and **will appear in the published article** if the submission is accepted. Please make sure it is accurate and that any funding sources listed in your Funding Information later in the submission form are also	NO authors have competing interests pertinent to this study.

declared in your Financial Disclosure statement.<br><br>View published research articles from *PLOS ONE* for specific examples.<br><br>**NO authors have competing interests**<br><br>Enter: *The authors have declared that no competing interests exist.*<br><br>**Authors with competing interests**<br><br>Enter competing interest details beginning with this statement:<br><br>*I have read the journal's policy and the authors of this manuscript have the following competing interests: [insert competing interests here]*<br><br>* typeset	
**Ethics Statement**<br><br>Enter an ethics statement for this submission. This statement is required if the study involved:<br><br>• Human participants<br>• Human specimens or tissue<br>• Vertebrate animals or cephalopods<br>• Vertebrate embryos or tissues<br>• Field research<br><br>Write "N/A" if the submission does not require an ethics statement.<br><br>General guidance is provided below. Consult the submission guidelines for detailed instructions. **Make sure that all information entered here is included in the Methods section of the manuscript.**	The University of Texas at Austin<br>2020-05-0040<br>Emails were sent to the participants and completing the surveys implied consent.

**Format for specific study types**<br><br>**Human Subject Research (involving human participants and/or tissue)**<br>• Give the name of the institutional review board or ethics committee that approved the study<br>• Include the approval number and/or a statement indicating approval of this research<br>• Indicate the form of consent obtained (written/oral) or the reason that consent was not obtained (e.g. the data were analyzed anonymously)<br><br>**Animal Research (involving vertebrate animals, embryos or tissues)**<br>• Provide the name of the Institutional Animal Care and Use Committee (IACUC) or other relevant ethics board that reviewed the study protocol, and indicate whether they approved this research or granted a formal waiver of ethical approval<br>• Include an approval number if one was obtained<br>• If the study involved *non-human primates*, add *additional details* about animal welfare and steps taken to ameliorate suffering<br>• If anesthesia, euthanasia, or any kind of animal sacrifice is part of the study, include briefly which substances and/or methods were applied<br><br>**Field Research**<br><br>Include the following details if this study involves the collection of plant, animal, or other materials from a natural setting:<br>• Field permit number<br>• Name of the institution or relevant body that granted permission	
**Data Availability**<br><br>Authors are required to make all data underlying the findings described fully available, without restriction, and from the time of publication. PLOS allows rare exceptions to address legal and ethical concerns. See the PLOS Data Policy and FAQ for detailed information.	No - some restrictions will apply

A Data Availability Statement describing where the data can be found is required at submission. Your answers to this question constitute the Data Availability Statement and **will be published in the article**, if accepted.<br><br>**Important:** Stating 'data available on request from the author' is not sufficient. If your data are only available upon request, select 'No' for the first question and explain your exceptional situation in the text box.<br><br>Do the authors confirm that all data underlying the findings described in their manuscript are fully available without restriction?	
**Describe where the data may be found in full sentences. If you are copying our sample text, replace any instances of XXX with the appropriate details.**<br><br>• If the data are **held or will be held in a public repository**, include URLs, accession numbers or DOIs. If this information will only be available after acceptance, indicate this by ticking the box below. For example: *All XXX files are available from the XXX database (accession number(s) XXX, XXX.*).<br>• If the data are all contained **within the manuscript and/or Supporting Information files**, enter the following: *All relevant data are within the manuscript and its Supporting Information files.*<br>• If neither of these applies but you are able to provide **details of access elsewhere**, with or without limitations, please do so. For example:<br><br>*Data cannot be shared publicly because of [XXX]. Data are available from the XXX Institutional Data Access / Ethics Committee (contact via XXX) for researchers who meet the criteria for access to confidential data.*<br><br>*The data underlying the results presented in the study are available from (include the name of the third party*	The data underlying the results presented in the study are available from Dr. David Ring.

and contact information or URL).<br>• This text is appropriate if the data are owned by a third party and authors do not have permission to share the data.<br><br>* typeset	
Additional data availability information:	

Powered by Editorial Manager® and ProduXion Manager® from Aries Systems Corporation

Manuscript

Click here to access/download;Manuscript;Manuscript Biomedical vs Biopych.docx

Musculoskeletal Surgeons Have Implicit Bias Towards the Biomedical Paradigm of Human Illness

[1]Sina Ramtin M.D[1]; Dayal Rajagopalan M.D.; [1]David Ring M.D.; Tom Crijns, MD; [1]Prakash Jayakumar, MD PhD;

[1]The University of Texas Dell Medical School, Austin, TX

Corresponding Author:

David Ring, MD PhD

The University of Texas Dell Medical School

1601 Trinity St. Building A. Austin TX 78712

Email: David.Ring@austin.utexas.edu

Phone Number: 512-495-3021

Conflicts of Interest and Source of Funding: None

SR, DR, D Ring, TC and PJ certify that they have no commercial associations (eg, consultancies, stock ownership, equity interest, patent/licensing arrangements, etc) that might pose a conflict of interest in connection with the submitted article. One of the authors (DR) received royalties from Skeletal Dynamics for an internal joint stabilizer elbow in the amount of between 10,000 and 100,000 USD per year. One of the authors certifies that he (DR) is a Deputy Editor for Hand and Wrist at Journal of Orthopaedic Trauma, and Clinical Orthopaedics and Related Research® and

has received or may receive payments or benefits in the amount of USD 5000 per year. One of the authors certifies that he (DR) received honoraria from various hospitals and universities.

Ethical Review Statement: This wok has been approved by our Institutional Review Board.

Source of Funding: None

Relevant financial disclosure outside the submitted work: board membership; research support, royalties, stock/shareholder, ownership, consultancy.

Acknowledgements: None

Keywords: implicit bias, surgeon bias, Implicit attitude test, biomedical, biopsychosocial

This work was performed at the University of Texas at Austin, Austin, Texas, USA

Abstract

Background: Evidence is mounting that the biopsychosocial paradigm is more accurate and useful than the biomedical paradigm of care. Habits of thought can hinder the implementation of this knowledge into daily care strategies. To understand and lessen these potential barriers, we asked: 1) What is the relative implicit and explicit attitudes of musculoskeletal surgeons towards the biomedical or biopsychosocial paradigms of medicine? 2) What surgeon factors are associated with these attitudes?

Methods: An online survey-based experiment was distributed to members of the Science of Variation Group (SOVG) with a total of 163 respondents. Implicit bias towards the biomedical or biopsychosocial paradigms was measured using an Implicit Association Test (IAT) developed by our team using open-source software; explicit preferences were measured using ordinal scales.

Results: On average, surgeons demonstrated a moderate implicit bias towards the biomedical paradigm (d-score: -0.21; Interquartile range [IQR]: -0.56 to 0.19) and a moderate explicit preference towards the biopsychosocial paradigm (mean: 14; standard deviation: 14). A greater implicit bias towards the biomedical paradigm was associated with male surgeons (d-score: -0.30; IQR: -0.57 to 0.14; *P* = 0.005). A greater explicit preference towards the biomedical paradigm was independently associated with a European practice location (Regression coefficient: -9.1; 95% CI: -14 to -4.4; *P* <0.001) and trauma subspecialty (RC: -6.2; 95% CI: -11 to -1.0; *P* <0.001).

Conclusions: The observation that surgeons have an implicit bias favoring the biomedical paradigm might inform strategies for implementation of care strategies based on evidence favoring the biopsychosocial paradigm.

Introduction

The biopsychosocial model posits that human illness results from the interplay of pathophysiology with thoughts, emotions, and circumstances[1–5]. The biomedical model of human illness considers symptoms as directly corresponding to underlying pathology[6,7]. The association between variation in mental health (e.g., unhelpful thoughts and unhelpful feelings) and social well-being (e.g., security in life roles, connectedness, housing, food, job, and finances) with variation in pain intensity and magnitude of incapability[5,8–12] relatively independent of pathology severity supports the utility of the biopsychosocial model[13–17]. Musculoskeletal specialists increasingly incorporate elements of the biopsychosocial model into daily practice[18], providing whole-person care that considers emotional needs and social circumstances, as well as the physical condition, while limiting low value tests and treatments[19]. Implementation of this line of evidence might be hindered by ingrained habits of thought and default actions (implicit bias) towards the biopsychosocial model.

Musculoskeletal specialists are well-positioned to discern the relative correspondence between pathophysiology and magnitude of incapability. There is compelling evidence that relatively greater magnitude of incapability signals mental health opportunities such as unhelpful thinking and feelings of worry or despair, along with social health opportunities such as insecurity in roles, finances, housing, and food [20,21]. Surgeon implicit bias contrary to this evidence could harm patients through missed opportunities to address mental and social health in combination with exposure to tests and treatments with more potential for harm than benefit.

Prior research suggests the presence of discordance between implicit and explicit attitudes for controversial or difficult subjects[22]. Presumably no surgeon would say they don't appreciate the importance of mental and social factors on their patients' overall health. Implicit

and explicit attitudes tend to use different modes of cognition (e.g., automatic/intuitive vs. critical/analytical)[22]. We might think of the explicit attitude as ~~the~~ aspiration and ~~the~~ implicit attitude as the starting point, with ~~the~~ difference between the two being the gap to be bridged to improve whole-person, comprehensive care.

Implicit attitude tests (IAT) can measure unconscious habits of thought such as a tendency to link obesity with laziness[23–27], or being a surgeon with being male[28]. Based on experience developing IATs to test implicit biases, we developed one to measure the implicit bias of musculoskeletal surgeons towards either ~~the~~ biomedical or biopsychosocial model of illness. Awareness of surgeon implicit bias towards the biomedical model might help promote debiasing strategies that support consideration of mental and social health in musculoskeletal care.

Therefore, we asked: What is the average relative implicit bias of musculoskeletal surgeons towards health as biomedical or biopsychosocial? 2) What is their relative explicit preference? 3) Which surgeon factors are associated with greater explicit preference or implicit bias towards either ~~the~~ biomedical or biopsychosocial paradigms? 4) Is there a correlation between ~~the~~ explicit preference and ~~the~~ implicit bias towards either paradigm?

Materials and Methods

Study Design and Setting

We obtained approval for this cross-sectional study from our Institutional Review Board. We created an online survey-based experiment (Qualtrics, Provo, Utah, USA) and distributed the survey among all members of the Science of Variation Group (SOVG) between February 1st and March 1st, 2021. The introduction to the survey stated explicitly that completing the survey

represented informed consent to use their anonymized data. No form of written or verbal consent was required by the institutional review board. The SOVG is an international consortium of musculoskeletal surgeons (including general orthopedic surgeons, hand surgeons and other specialists, and fracture surgeons [often general surgeons in Europe]) who participate in monthly survey-based experiments to better understand variations in musculoskeletal care. The experiments conducted using the SOVG rely on within-sample variation to find associations. There is sufficient variation in survey responses among participants that the associations are likely reproducible. Take for instance the factors associated with implicit attitudes. The absolute rates, like mean scores for explicit and implicit attitudes, are likely not reproducible. The SOVG primarily consists of orthopedic surgeons who are white, male, academic, and based in the US or Europe. The respondent demographics are not by design but reflect the current lack of diversity in the field. We welcome new members and collaborators (https://www.surveymonkey.com/r/SOVG_FB). Our survey-based experiment was written in English and consisted of two components designed to 1) measure implicit biases using an implicit association test, and 2) to measure explicit attitudes and preferences for either ~~the~~ biomedical or biopsychosocial model on slider scales. We administered the explicit measure at the end to avoid priming respondents before doing the IAT.

There are about two hundred SOVG surgeons that participate in at least one survey experiment per year. One hundred and sixty-three surgeons completed this experiment. The majority were men (n=141, 90%), and practice in the United States (n=86, 53%) or Europe (n=52, 32%). More than half of surgeons had 11 or more years of experience (n=96, 61%), were fellowship-trained in trauma (n= 62, 40%) or hand surgery (n=51, 33%) (Table 1). While the gender distribution of our study demographic is representative of the field of orthopedic surgery

(around 90% men) it may not adequately represent associations of gender with implicit or explicit bias.

Implicit Association Test (IAT)

An IAT is based on the premise that the longer it takes a respondent to associate a concept with positive descriptors, the more the concept is contrary to current habits of thought (implicit bias), and vice versa[29]. The IAT works by showing a concept on screen along with a descriptive category on each side (right and left). Someone taking the IAT can press a button on their keyboard that sorts the concept into either ~~the~~ right or ~~the~~ left category. The speed and accuracy with which respondents sort the concept into a category over a series of blocks is distilled into a single score, the d-score. The conventional d-score calculation involves dropping the first two trials in each testing block (blocks 4 and 7), recoding trial latencies outside of the established bounds (300 milliseconds to 3000 milliseconds) to being equivalent to the closest bound, log transforming the latency values before averaging them, including latency values from trials that were answered incorrectly, and finally omitting altogether data from respondents whose latencies are far higher than what is expected for the subject being analyzed[22].

The utility and validity of IATs has a strong basis in prior studies, and IATs have been used in a wide range of topics to measure implicit cognitive biases[30–32]. We created an IAT using IATgen to test musculoskeletal surgeons' bias toward ~~the~~ biomedical or biopsychosocial paradigms[33]. Through a working group discussion, we created a list of concept terms (words that are associated with either ~~the~~ biomedical or biopsychosocial paradigm) and attribute terms (words that have a positive or negative connotation) (Table 2). The authors selected terms we thought were a) representative of the concept we wanted to test and b) universal, so that

experiment participants around the world would understand them. Terms were introduced by one team member and adjusted/approved by the others until consensus was reached. The working group consisted of multiple orthopedic surgeons and orthopedic research fellows. The IAT was conducted as outlined by Greenwald et al. and consisted of 7 association blocks. Blocks 1, 2, 3, 5, and 6 were for teaching, while blocks 4 and 7 were for evaluation (Table 3)[34,35]. Blocks 1, 2, 3, 5, and 6 were essentially "practice" blocks that were not scored and were in place to help respondents acclimate to the IAT itself before they completed blocks that were scored. The IAT is meant to test automatic or intuitive thinking, but this works best when respondents are fully acclimated to the format of the test. Participants are asked to sort the concept terms with the attribute terms as quickly as possible. During a testing block, we recorded the time needed to make an association to the millisecond. There are 4 permutations of an IAT based on the order and handedness (left vs. right) and surgeons were randomized into one of these 4 groups.

Measured variables

Our primary outcome of interest was the d-score of the IAT, which is calculated based on the time it takes an observer to associate certain concepts, and ranges from -1.5 to 1.5. A d-score of 0 indicates no bias in either direction while a d-score of greater magnitude indicates greater implicit bias towards one habit of thinking. In this context, a more negative d-score indicates a stronger bias towards the biomedical paradigm. In the questionnaire portion of the survey, surgeons were asked 4 questions to gauge explicit preference for either the biomedical or biopsychosocial paradigm, rated on an 11-point ordinal scale (table 6). Surgeons' explicit preference was converted to a total score on a scale from -50 to 50, with more positive scores indicating a stronger explicit preference for the biopsychosocial paradigm. We converted the 11-

point ordinal scale to a scale ranging from -50 to +50 to make the presentation of the results more intuitive. For instance, whole numbers rather than decimals.

Statistical analysis

Descriptive statistics were performed of all participants. Discrete variables were reported as number (percentage); continuous variables as mean ± standard deviation or median (interquartile range). Mean was used when ~~the~~ distribution of the variable was normal while median was used when the variable was non-normal or had significant skew. We did not do ~~an~~ a priori power analysis because we had a fixed pool of respondents to draw from. Shapiro-Wilk tests were used to assess normality. Explicit bias scores were normally distributed while implicit bias scores were not. Student t-tests were used when analyzing relationships between explicit bias scores and binary variables. One-way analysis of variance (ANOVA) tests were used to analyze relationships between explicit bias scores and non-binary variables. Wilcoxon rank sum tests were used when analyzing relationships between implicit bias scores and binary variables. Kruskal-Wallis H tests were used for analyzing the relationships between implicit bias scores and non-binary variables. In multivariable analysis, the response variable was explicit preference for the biopsychosocial paradigm. The explanatory variables were practice location (continent) and orthopedic subspecialty. All explanatory variables with $P < 0.10$ in bivariate analysis were moved to multivariable linear regression analysis[36]. A single regression model was created with qualifying variables (practice location and subspecialty). Regression coefficients (RC), 95% Confidence Intervals (95% CI), standard errors, *P*-values, partial r-squared, and adjusted r-squared values were reported.

Results

Questions 1 and 2: What is the average relative implicit bias of musculoskeletal surgeons towards health as biomedical or biopsychosocial? What is their relative explicit preference?

On average, surgeons reported an explicit preference for the biopsychosocial model (mean: 14; standard deviation: 14) (Figure 1) but had an implicit bias favoring the biomedical paradigm (d-score: -0.21; interquartile range [IQR]: -0.56 to 0.19) (Figure 2).

Question 3: Which surgeon factors are associated with greater explicit preference or implicit bias towards either ~~the~~ biomedical or biopsychosocial paradigms?

In bivariate analysis, men had a greater implicit bias favoring the biomedical paradigm (d-score: -0.30; IQR: -0.57 to 0.14) than women (d-score: 0.12; IQR: -0.23 to 0.65) (*P* = 0.005; Table 4). There were no other surgeon factors associated with implicit bias, and no multivariable analysis was performed.

Accounting for potential confounders in multivariable analysis, a greater explicit preference towards the biomedical paradigm was independently associated with a European practice location (Regression coefficient: -9.1; 95% CI: -14 to -4.4; *P* <0.001), and trauma as a subspecialty (RC: -6.2; 95% CI: -11 to -1.0; *P* <0.001) (Table 5).

Question 4: Is there a correlation between ~~the~~ explicit preference and ~~the~~ implicit bias towards either paradigm?

There was no correlation between ~~the~~ extent of explicit preference for, or implicit bias towards either paradigm (ρ= 0.10; *P* = 0.19; Figure 3).

Discussion

The notable variability in illness severity for a given pathology cannot be fully explained in the biomedical paradigm of human illness [12,16]. Implementation of ~~the~~ evidence that mental and social factors have a notable association with symptom intensity[12,37–40] into daily practice might be hindered by habits of thought and action (unconscious bias) favoring ~~the~~ biomedical relative to ~~the~~ biopsychosocial model. We conducted a survey-based experiment of members of the Science of Variation Group and found that musculoskeletal surgeons reported an explicit preference for the biopsychosocial paradigm and had an implicit bias favoring the biomedical paradigm.

This study had several limitations. First, some words used in ~~the~~ IAT (e.g., therapy) are somewhat ambiguous, and may be interpreted variably (e.g., ‘physical therapy’ rather than ‘mental health counseling’). In addition, our participants are an international group of surgeons, some of whom speak English as a second language. The training rounds support participants with ~~the~~ intended meaning and associations of these words, which should mitigate the effects of English dialect and proficiency. Second, the explicit preference was measured after ~~the~~ IAT, which could skew the explicit preference. However, there is evidence that the order has relatively little influence on the results[32]. Finally, the SOVG largely consists of white men in academic practice. The comparison of explicit preference between men and women, and between surgeons who do or do not supervise trainees could be relatively underpowered. However, we believe that differences between these individual subgroups are less generalizable and arguably less important than the finding of discordance between explicit preference and implicit bias towards the biomedical model. Additionally, the SOVG may tend to attract participation of surgeons who are interested in the subject-matter at hand, and this may constitute a form of selection bias. The

observation of a relative implicit bias that health is biomedical, contrary to the expressed preference for the biopsychosocial paradigm, is notable in the subpopulation of the SOVG that chose to participate. The relative implicit bias could be even greater among other surgeons. Importantly, the part of the experiment measuring associations relies on within-sample variation to find associations. These findings might be relatively reproducible in samples with different mean implicit and explicit biases if there is sufficient within sample variation.

The finding that, on average, surgeons have an implicit relative biomedical bias and an explicit relative biopsychosocial preference demonstrates the ongoing transition towards whole-person models of care that anticipates the influence of mood, coping strategies, and hindrance of life roles on illness severity and recovery trajectories[39,41]. In other words, surgeons understand the utility of the biopsychosocial model but are still working to evolve habits of thinking that unconsciously favor the biomedical model. The shift over the last decade from clinician-reported measures (for example, the Mayo Elbow Performance Index[42] and the Musculoskeletal Tumor Society MSTS scoring system[43]) to patient-rated measures of capability (e.g., Disabilities of the Arm, Shoulder and Hand [DASH] questionnaire[44] and the Patient-Reported Outcomes Measurement Information System [PROMIS] Physical Function)[45] illustrates the increasing interest in gauging health from the patient's perspective.

A variety of evidence-based strategies exist to mitigate bias[28]. Clinicians can acknowledge and affirm uncomfortable thoughts rather than suppressing them[46]. They can be conscientious of their gut feelings during certain situations. After recognizing uncomfortable thoughts and gut feelings, clinicians can consider changing settings and situations that may give rise to negative or stereotypical responses[47]. These may seem like "common sense" interventions, but they are difficult to implement in real-time, especially in high-pressure clinical

situations. Specific training from experts may ease the implementation of these strategies, particularly during malleable periods of training, such as residency or medical school. Individual-level interventions can be paired with system-level interventions to achieve meaningful changes. Policymakers can promote the biopsychosocial model by affirming it as part of core organizational and institutional values[48]. Additionally, policymakers and leaders can implement systems that promote ~~the~~ anticipation and detection of bias. They can respond to bias detection by changing institutions that propagate those biases[49].

The observations that a surgeon factor (gender) was associated with the degree of implicit bias, ~~and~~ practice location and subspecialty were associated with the degree of explicit preference, demonstrates the influence of varied experience, and has implications about the ability to cultivate new automatic thoughts about the biopsychosocial paradigm. Bias training, when performed effectively and administered to the correct target groups, can effectively improve the existence of harmful implicit and explicit biases[50–52]. Our findings that bias against the biopsychosocial paradigm may be associated with certain subgroups within the orthopedic surgery community may allow for targeting of those subgroups for effective elimination of those biases. There is little to gain from providing bias elimination training to those who do not harbor that bias[52].

The observation that there was no correlation between surgeon explicit preference and implicit bias for the biopsychosocial model is in line with prior research, suggesting that explicit preference and implicit bias are subject to different cognitive forces[53,54]. Given that most of us received medical training embedded in the biomedical paradigm and given that this is still the paradigm in which most musculoskeletal care is designed and delivered, this mismatch between explicit preference and implicit bias is expected. The mismatch may also be due to ~~the~~ social

desirability bias[55,56] given that all surgeons in the database are familiar with the research by the senior authors, and it is possible that they gave socially desirable answers when asked directly about their interest in biopsychosocial research.

We performed an implicit association test among an international group of surgeons and found that most have an explicit relative preference for ~~the~~ biopsychosocial model and an implicit relative bias in favor of ~~the~~ biomedical paradigm. Implicit association testing has the potential to enhance surgeon self-awareness, professional and personal development, and quality improvement efforts. Greater surgeon awareness of implicit biases can aid the transition towards comprehensive care models that acknowledge the influence of environmental, mental, and social factors on illness severity. Future studies may measure the impact of biases towards ~~the~~ biomedical paradigm on surgeon participation in comprehensive care models.

References

1. Engel GL. The Biopsychosocial Model and Medical Education. New England Journal of Medicine. 1982;306: 802–805. doi:10.1056/NEJM198204013061311

2. Engel GL. From Biomedical to Biopsychosocial: Being Scientific in the Human Domain. Psychosomatics. 1997;38: 521–528. doi:https://doi.org/10.1016/S0033-3182(97)71396-3

3. Engel GL. THE BIOPSYCHOSOCIAL MODEL AND THE EDUCATION OF HEALTH PROFESSIONALS*†. Ann N Y Acad Sci. 1978;310: 169–181. doi:https://doi.org/10.1111/j.1749-6632.1978.tb22070.x

4. Engel GL. The Clinical Application of the Biopsychosocial Model. The Journal of Medicine and Philosophy: A Forum for Bioethics and Philosophy of Medicine. 1981;6: 101–124. doi:10.1093/jmp/6.2.101

5. Wade DT, Halligan PW. The biopsychosocial model of illness: a model whose time has come. Clin Rehabil. 2017;31: 995–1004. doi:10.1177/0269215517709890

6. Burns CR. History in medical education: the development of current trends in the United States. Bulletin of the New York Academy of Medicine: Journal of Urban Health. 1975.

7. Wade DT, Halligan PW. Do biomedical models of illness make for good healthcare systems? BMJ. 2004;329: 1398 LP – 1401. doi:10.1136/bmj.329.7479.1398

8. Teunis T, Bot AGJ, Thornton ER, Ring D. Catastrophic Thinking Is Associated With Finger Stiffness After Distal Radius Fracture Surgery. J Orthop Trauma. 2015;29: e414-20. doi:10.1097/BOT.0000000000000342

9. Bowes SM, Ammirati RJ, Costello TH, Basterfield C, Lilienfeld SO. Cognitive biases, heuristics, and logical fallacies in clinical practice: A brief field guide for practicing clinicians and supervisors. Professional Psychology: Research and Practice. Bowes, Shauna M.: Department of

Psychology, Emory University, 36 Eagle Row, Atlanta, GA, US, 30322, shauna.m.bowes@gmail.com: American Psychological Association; 2020. pp. 435–445. doi:10.1037/pro0000309

10. Evans S, Okoroafor UC, Calfee RP. Is Social Deprivation Associated with PROMIS Outcomes After Upper Extremity Fractures in Children? Clin Orthop Relat Res. 2021;479: 826–834. doi:10.1097/CORR.0000000000001571

11. Wright MA, Beleckas CM, Calfee RP. Mental and Physical Health Disparities in Patients With Carpal Tunnel Syndrome Living With High Levels of Social Deprivation. J Hand Surg Am. 2019;44: 335.e1-335.e9. doi:10.1016/j.jhsa.2018.05.019

12. Kim KW, Han JW, Cho HJ, Chang CB, Park JH, Lee JJ, et al. Association between comorbid depression and osteoarthritis symptom severity in patients with knee osteoarthritis. Journal of Bone and Joint Surgery - Series A. 2011. doi:10.2106/JBJS.I.01344

13. Cremers T, Zoulfi Khatiri M, van Maren K, Ring D, Teunis T, Fatehi A. Moderators and Mediators of Activity Intolerance Related to Pain. J Bone Joint Surg Am. 2021;103: 205–212. doi:10.2106/JBJS.20.00241

14. Jayakumar P, Teunis T, Vranceanu A-M, Moore MG, Williams M, Lamb S, et al. Psychosocial factors affecting variation in patient-reported outcomes after elbow fractures. J Shoulder Elbow Surg. 2019;28: 1431–1440. doi:10.1016/j.jse.2019.04.045

15. Crijns TJ, Bernstein DN, Teunis T, Gonzalez RM, Wilbur D, Ring D, et al. The Association Between Symptoms of Depression and Office Visits in Patients With Nontraumatic Upper-Extremity Illness. Journal of Hand Surgery. 2020. doi:10.1016/j.jhsa.2019.03.019

16. Briet JP, Houwert RM, Hageman MGJS, Hietbrink F, Ring DC, Verleisdonk EJJM. Factors associated with pain intensity and physical limitations after lateral ankle sprains. Injury. 2016;47: 2565–2569. doi:10.1016/j.injury.2016.09.016

17. Kim KW, Han JW, Cho HJ, Chang CB, Park JH, Lee JJ, et al. Association between comorbid depression and osteoarthritis symptom severity in patients with knee osteoarthritis. Journal of Bone and Joint Surgery - Series A. 2011. doi:10.2106/JBJS.I.01344

18. Jayakumar P, Overbeek CL, Lamb S. What Factors Are Associated With Disability After Upper Extremity Injuries ? A Systematic Review. 2018. doi:10.1097/CORR.0000000000000427

19. Emery DJ, Shojania KG, Forster AJ, Mojaverian N, Feasby TE. Overuse of Magnetic Resonance Imaging. JAMA Intern Med. 2013;173: 823–825. doi:10.1001/jamainternmed.2013.3804

20. Ring D. Priorities for Advancing Mental and Social Health Among People Presenting for Care of Musculoskeletal Symptoms : International Consortium for Mental and Social Health in Musculoskeletal Care. J Clin Psychol Med Settings. 2022. doi:10.1007/s10880-022-09865-w

21. Vranceanu A-M, Bakhshaie J, Reichman M, Ring D, (IMESH) O behalf of TIMM and SHC. A Call for Interdisciplinary Collaboration to Promote Musculoskeletal Health: The Creation of the International Musculoskeletal Mental and Social Health Consortium (I-MESH). J Clin Psychol Med Settings. 2021. doi:10.1007/s10880-021-09827-8

22. Kahneman D. Thinking, fast and slow. Thinking, fast and slow. New York, NY, US: Farrar, Straus and Giroux; 2011. p. 499.

23. Wang SS, Brownell KD, Wadden TA. The influence of the stigma of obesity on overweight individuals. Int J Obes. 2004;28: 1333–1337. doi:10.1038/sj.ijo.0802730

24. Chambliss HO, Finley CE, Blair SN. Attitudes toward Obese Individuals among Exercise Science Students. Medicine & Science in Sports & Exercise. Chambliss, Heather O.: Cooper Institute,

12330 Preston Road, Dallas, TX, US, 75230, hchamblis@cooperinst.org: Lippincott Williams & Wilkins; 2004. pp. 468–474. doi:10.1249/01.MSS.0000117115.94062.E4

25. Robertson N, Vohora R. Fitness vs. fatness: Implicit bias towards obesity among fitness professionals and regular exercisers. Psychol Sport Exerc. 2008;9: 547–557. doi:https://doi.org/10.1016/j.psychsport.2007.06.002

26. Schwartz MB, Chambliss HO, Brownell KD, Blair SN, Billington C. Weight Bias among Health Professionals Specializing in Obesity. Obes Res. 2003;11: 1033–1039. doi:https://doi.org/10.1038/oby.2003.142

27. Teachman BA, Brownell KD. Implicit anti-fat bias among health professionals: is anyone immune? Int J Obes. 2001;25: 1525–1531. doi:10.1038/sj.ijo.0801745

28. Salles A, Awad M, Goldin L, Krus K, Lee JV, Schwabe MT, et al. Estimating Implicit and Explicit Gender Bias Among Health Care Professionals and Surgeons. JAMA Netw Open. 2019;2: e196545–e196545. doi:10.1001/jamanetworkopen.2019.6545

29. Greenwald AG, McGhee DE, Schwartz JLK. Measuring individual differences in implicit cognition: the implicit association test. J Pers Soc Psychol. 1998;74: 1464.

30. Greenwald AG, Poehlman TA, Uhlmann EL, Banaji MR. Understanding and using the Implicit Association Test: III. Meta-analysis of predictive validity. Journal of Personality and Social Psychology. Greenwald, Anthony G.: Department of Psychology, University of Washington, Box 351525, Seattle, WA, US, 98195-1525, agg@u.washington.edu: American Psychological Association; 2009. pp. 17–41. doi:10.1037/a0015575

31. Nosek BA, Smyth FL. A Multitrait-Multimethod Validation of the Implicit Association Test. Exp Psychol. 2007;54: 14–29. doi:10.1027/1618-3169.54.1.14

32. Nosek BA, Greenwald AG, Banaji MR. Understanding and Using the Implicit Association Test: II. Method Variables and Construct Validity. Pers Soc Psychol Bull. 2005;31: 166–180. doi:10.1177/0146167204271418

33. Carpenter TP, Pogacar R, Pullig C, Kouril M, Aguilar S, LaBouff J, et al. Survey-software implicit association tests: A methodological and empirical analysis. Behav Res Methods. 2019;51: 2194–2208. doi:10.3758/s13428-019-01293-3

34. Lane KA, Banaji MR, Nosek BA, Greenwald AG, Wittenbrink B, Schwarz N. Implicit measures of attitudes. Understanding and Using the Implicit Association Test: IV. 2007; 59–102.

35. Greenwald AG, McGhee DE, Schwartz JLK. Measuring individual differences in implicit cognition: the implicit association test. J Pers Soc Psychol. 1998;74: 1464.

36. Westfall PH, Young SS. Resampling-based multiple testing: Examples and methods for p-value adjustment. John Wiley & Sons; 1993.

37. Bernstein DN, Crijns TJ, Mahmood B, Ring D, Hammert WC. Patient Characteristics, Treatment, and Presenting PROMIS Scores Associated with Number of Office Visits for Traumatic Hand and Wrist Conditions. Clin Orthop Relat Res. 2019;477. doi:10.1097/CORR.0000000000000742

38. Crijns TJ, Bernstein DN, Ring D, Gonzalez R, Wilbur D, Hammert WC. Factors Associated With a Discretionary Upper-Extremity Surgery. Journal of Hand Surgery. 2019;44. doi:10.1016/j.jhsa.2018.04.028

39. Jayakumar P, Overbeek CL, Lamb S. What Factors Are Associated With Disability After Upper Extremity Injuries ? A Systematic Review. 2018. doi:10.1097/CORR.0000000000000427

40. Jayakumar P, Teunis T, Vranceanu A-M, Moore MG, Williams M, Lamb S, et al. Psychosocial factors affecting variation in patient-reported outcomes after elbow fractures. J Shoulder Elbow Surg. 2019;28: 1431–1440. doi:10.1016/j.jse.2019.04.045

41. Jayakumar P, Teunis T, Vranceanu AM, Lamb S, Ring D, Gwilym S. Early Psychological and Social Factors Explain the Recovery Trajectory After Distal Radial Fracture. J Bone Joint Surg Am. 2020;102: 788–795. doi:10.2106/JBJS.19.00100

42. Cusick MC, Bonnaig NS, Azar FM, Mauck BM, Smith RA, Throckmorton TW. Accuracy and reliability of the Mayo Elbow Performance Score. J Hand Surg Am. 2014;39: 1146–1150. doi:10.1016/j.jhsa.2014.01.041

43. Janssen SJ, Van Rein EAJ, Paulino Pereira NR, Raskin KA, Ferrone ML, Hornicek FJ, et al. The Discrepancy between Patient and Clinician Reported Function in Extremity Bone Metastases. Sarcoma. 2016. doi:10.1155/2016/1014248

44. Gummesson C, Atroshi I, Ekdahl C. The disabilities of the arm, shoulder and hand (DASH) outcome questionnaire: longitudinal construct validity and measuring self-rated health change after surgery. BMC Musculoskelet Disord. 2003;4: 11. doi:10.1186/1471-2474-4-11

45. Rothrock NE, Kaat AJ, Vrahas MS, O'Toole R V, Buono SK, Morrison S, et al. Validation of PROMIS Physical Function Instruments in Patients With an Orthopaedic Trauma to a Lower Extremity. J Orthop Trauma. 2019;33: 377–383. doi:10.1097/BOT.0000000000001493

46. Ranganath KA, Smith CT, Nosek BA. Distinguishing automatic and controlled components of attitudes from direct and indirect measurement methods. J Exp Soc Psychol. 2008;44: 386–396.

47. Barden J, Maddux WW, Petty RE, Brewer MB. Contextual moderation of racial bias: the impact of social roles on controlled and automatically activated attitudes. J Pers Soc Psychol. 2004;87: 5.

48. Moskowitz GB. On the control over stereotype activation and stereotype inhibition. Soc Personal Psychol Compass. 2010;4: 140–158.

49. Banaji MR, Bhaskar R. Implicit stereotypes and memory: The bounded rationality of social beliefs. Memory, brain, and belief. 2000; 139–175.

50. Stone J, Moskowitz GB. Non-conscious bias in medical decision making: what can be done to reduce it? Med Educ. 2011;45: 768–776. doi:https://doi.org/10.1111/j.1365-2923.2011.04026.x

51. Betancourt JR, Ananeh-Firempong O. Not me! Doctors, decisions, and disparities in health care. Cardiovasc Rev Rep. 2004;25: 105–109.

52. Jackson SM, Hillard AL, Schneider TR. Using implicit bias training to improve attitudes toward women in STEM. Social Psychology of Education. 2014;17: 419–438. doi:10.1007/s11218-014-9259-5

53. Hofmann W, Gawronski B, Gschwendner T, Le H, Schmitt M. A meta-analysis on the correlation between the implicit association test and explicit self-report measures. Pers Soc Psychol Bull. 2005;31: 1369–1385. doi:10.1177/0146167205275613

54. Sabin J, Nosek BA, Greenwald A, Rivara FP. Physicians' implicit and explicit attitudes about race by MD race, ethnicity, and gender. J Health Care Poor Underserved. 2009;20: 896–913. doi:10.1353/hpu.0.0185

55. Rao A, Tobin K, Davey-Rothwell M, Latkin CA. Social Desirability Bias and Prevalence of Sexual HIV Risk Behaviors Among People Who Use Drugs in Baltimore, Maryland: Implications for Identifying Individuals Prone to Underreporting Sexual Risk Behaviors. AIDS Behav. 2017;21: 2207–2214. doi:10.1007/s10461-017-1792-8

56. Widmar NJO, Byrd ES, Dominick SR, Wolf CA, Acharya L. Social desirability bias in reporting of holiday season healthfulness. Prev Med Rep. 2016;4: 270–276. doi:10.1016/j.pmedr.2016.06.017

Figure legends:

Figure 1: Surgeons' explicit preference for the biomedical or biopsychosocial paradigm, scored on a scale from -50 to 50.

Figure 2: Surgeons' implicit preference for the biomedical or biopsychosocial paradigm, scored on a scale from -1.5 to 1.5.

Figure 3: Scatter plot of the implicit and explicit surgeon preferences for the biomedical or biopsychosocial paradigm.

Click here to access/download;Figure;Figure 1.docx

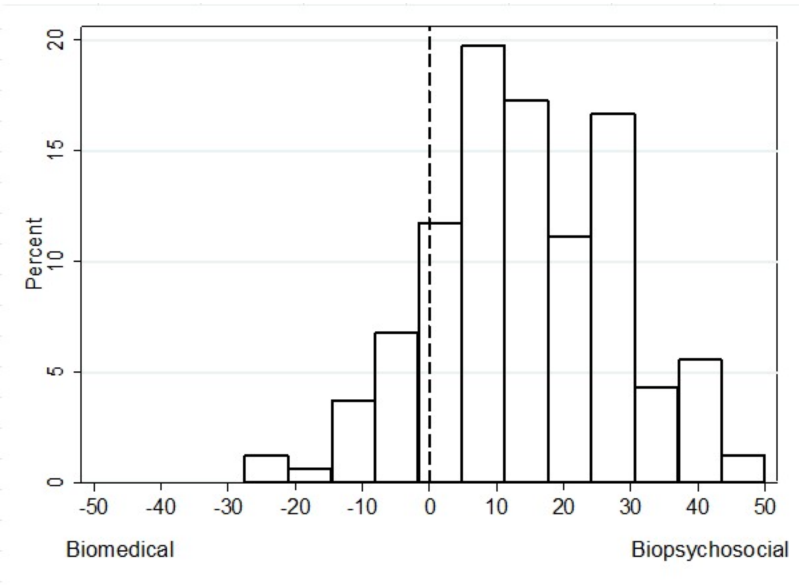

Click here to access/download;Figure;Figure 2.docx

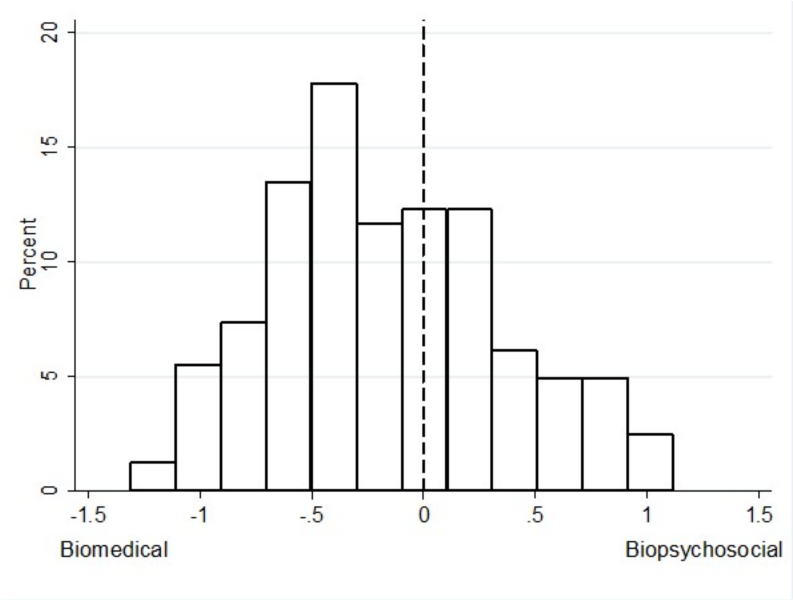

Click here to access/download;Figure;Figure 3.docx

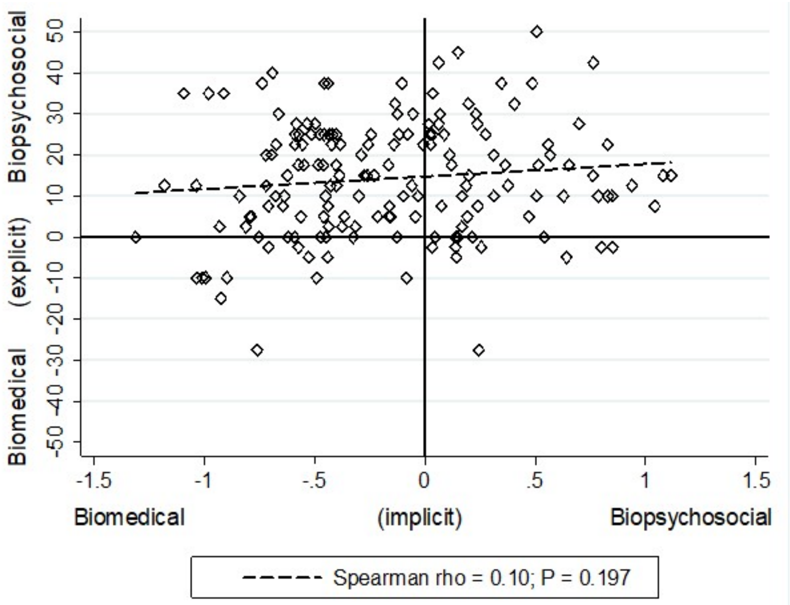

Click here to access/download
Supporting Information
_table_1.docx

Click here to access/download
Supporting Information
_table_2.docx

Click here to access/download
Supporting Information
_table_3.docx

Click here to access/download
Supporting Information
_table_4.docx

Click here to access/download
Supporting Information
_table_5.docx

Click here to access/download
Supporting Information
_table_6.docx
