# Supplementary material for: Musculoskeletal surgeons have implicit bias towards the biomedical paradigm of human illness
Source: PLoS One. 2024 Oct 24;19(10):e0310119. doi: 10.1371/journal.pone.0310119 (PMC11500957; doi:10.1371/journal.pone.0310119)
Supplement: Attachment — Submitted filename: Response to Reviewers.docx [file pone.0310119.s003.docx]

**Response to Reviewer Comments**

**Reviewer #1:**

1. Methods: Line 117 - consider removing "We welcome new members and collaborators"

**Response/Changes:** Removed.

2. Is there any reason why the relationship between years of experience and implicit bias or explicit preference was not explored?

**Response/Changes:** The relationship of years of experience and implicit bias was addressed (see Table 4) and no significant association was found.

3. Authors should provide the test-retest reliability and internal consistency values of the IAT

**Response/Changes:** These two values are not related to this study. Those are mainly used in the meta-analysis of IAT scores. Therefore, those are not applied here.

**Reviewer #2:** 
1. Few adjustments must be adhered to which has been highlighted in the corrected manuscript.

**Response/Changes:** All the comment in the manuscript were addressed.
